# Coronary Ectasia and Ventricular Arrhythmia in a Patient With Prior Tick Exposure and Borrelia burgdorferi IgG Seroreactivity: A Case Report

**DOI:** 10.7759/cureus.114666

**Published:** 2026-08-17

**Authors:** Rozen K Grigorov, Radosveta I Angelova, Stefan N Yambolov, Yanko G Yankov

**Affiliations:** 1 Department of Cardiology and Rheumatology, Medical University “Prof. Dr. Paraskev Stoyanov”, Varna, BGR; 2 First Department of Internal Medicine, University Hospital “St. Marina”, Varna, BGR; 3 Clinic of Maxillofacial Surgery, University Hospital “St. Marina”, Varna, BGR; 4 Department of General and Operative Surgery, Medical University “Prof. Dr. Paraskev Stoyanov”, Varna, BGR

**Keywords:** borrelia burgdorferi, case report, coronary ectasia, coronary vasculitis, lyme carditis, lyme disease, non-obstructive coronary artery disease, ranolazine

## Abstract

Lyme borreliosis is a multisystem infectious disease caused by *Borrelia burgdorferi* and transmitted to humans through the bite of infected *Ixodes* ticks that most commonly affects the skin, joints, nervous system, and, less frequently, the heart. Lyme carditis typically presents with atrioventricular conduction abnormalities, whereas coronary vascular involvement is rarely described.

We report a case of a 74-year-old man with exertional chest discomfort, dyspnea, and palpitations several months after an untreated tick bite. Electrocardiography demonstrated ventricular bigeminy with repolarization abnormalities, and 24-hour Holter monitoring showed a high burden of polymorphic premature ventricular complexes, with one episode of non-sustained ventricular tachycardia. Echocardiography demonstrated preserved left ventricular systolic function. Serological testing showed positive anti-*B. burgdorferi* IgG antibodies and negative IgM antibodies, consistent with prior exposure. Invasive coronary angiography revealed no obstructive epicardial coronary artery disease but demonstrated marked ectasia of the left anterior descending and right coronary arteries, both measuring up to 6.5 mm, with prolonged mural contrast retention. In the setting of exertional angina, persistent contrast retention within ectatic coronary segments, frequent ventricular ectopy, baseline bradycardia, and QTc prolongation, ranolazine was initiated as an antianginal therapy with potential antiarrhythmic benefit. At one-month follow-up, the patient reported marked improvement in symptoms. Repeat Holter monitoring showed a reduction in premature ventricular complexes to less than 5%, with no further episodes of non-sustained ventricular tachycardia.

This case suggests that, in patients with coronary ectasia and a history compatible with prior untreated *B. burgdorferi* infection, Lyme-associated coronary vascular injury may be considered as a possible underlying mechanism, although definitive causality remains difficult to establish. Coronary ectasia with prolonged contrast retention should be recognized as a potential substrate for myocardial ischemia and ventricular arrhythmogenesis in patients without obstructive coronary artery disease. In selected patients with anginal symptoms and frequent ventricular ectopy, particularly when beta-blockers or conventional antiarrhythmic drugs are limited by bradycardia, QTc prolongation, or concern for conduction disease, ranolazine may offer a useful therapeutic option with combined antianginal and potential antiarrhythmic benefit.

## Introduction

Lyme borreliosis is a multisystem infectious disease caused by *Borrelia burgdorferi* and transmitted to humans through the bite of infected *Ixodes* ticks. In Bulgaria, it is the most frequently reported tick-borne infection, with an average annual incidence of 3.64 cases per 100,000 population [1]. Its clinical manifestations include dermatologic, rheumatologic, and, less commonly, neurologic and cardiac complications [2,3]. The most frequent cardiac manifestation is Lyme carditis, which is typically associated with conduction system abnormalities, particularly atrioventricular block ranging from first-degree to complete heart block, although this is usually transient [4]. This process is thought to result from an immune-mediated inflammatory response triggered by myocardial invasion of *Borrelia* organisms [5].

Although uncommon, Lyme infection may involve the vascular system through inflammatory injury of the vessel wall, resulting in vasculitic complications. Cerebral vascular involvement has been reported in association with ischemic stroke, intracranial aneurysms, and carotid artery aneurysms [6,7]. The proposed mechanisms include direct vascular invasion by *B. burgdorferi*, immune-mediated endothelial injury, or a combination of both processes [8]. Such vascular injury may promote endothelial dysfunction and vessel-wall remodeling, providing a plausible mechanism for coronary aneurysmal dilatation or ectasia. Coronary vascular involvement related to Lyme disease has been described only in isolated reports and may manifest with anginal symptoms and coronary aneurysmal dilatation on invasive coronary angiography [9,10]. In addition, rare reports of Lyme carditis with suspected ischemic involvement further support the potential overlap between Lyme-associated cardiac inflammation and coronary ischemic syndromes [11]. Although clinical reports of coronary involvement remain exceptional, pathological studies in cases of sudden cardiac death associated with Lyme carditis have demonstrated perivascular lymphocytic infiltration and spirochetes within cardiac tissue, supporting a potential vascular inflammatory component [12].

We present the case of a 74-year-old man with anginal symptoms and ventricular arrhythmia in the setting of prior tick exposure and positive *B. burgdorferi* serology. Coronary angiography showed non-obstructive coronary arteries with severe coronary ectasia and marked contrast retention, suggesting post-infectious coronary vascular involvement as a potential substrate for myocardial ischemia and ventricular arrhythmia. Ranolazine and doxycycline therapy were followed by clinical improvement and a substantial reduction in ventricular ectopic burden.

## Case presentation

A 74-year-old man was admitted to the Cardiology Department with progressive exertional chest discomfort, dyspnea, and palpitations. The symptoms had gradually worsened over several months and were accompanied by reduced exercise tolerance and fatigue during routine daily activities. His medical history included arterial hypertension, benign prostatic hyperplasia, and impaired glucose tolerance, with no previously documented obstructive coronary artery disease. Molsidomine had been initiated empirically because of the recently developed exertional chest discomfort while the patient was awaiting further cardiological evaluation and subsequent hospital referral. He was also on rosuvastatin 10 mg once daily. The patient was a non-smoker, with a body mass index of 31 kg/m² and no relevant family history of cardiovascular disease. He had no previous history of angina and had not undergone prior coronary imaging.

Eight months before admission, the patient recalled a tick bite followed several days later by an erythematous lesion at the site of the bite. The description was consistent with erythema migrans; however, no photograph or medical documentation was available. The patient did not seek medical attention at that time and received no antibiotic therapy. On admission, he was clinically stable. Blood pressure was 120/70 mmHg, heart rate was 60 beats/min, and the rhythm was regular. There were no signs of pulmonary congestion, peripheral edema, or overt heart failure.

**Figure 1:**
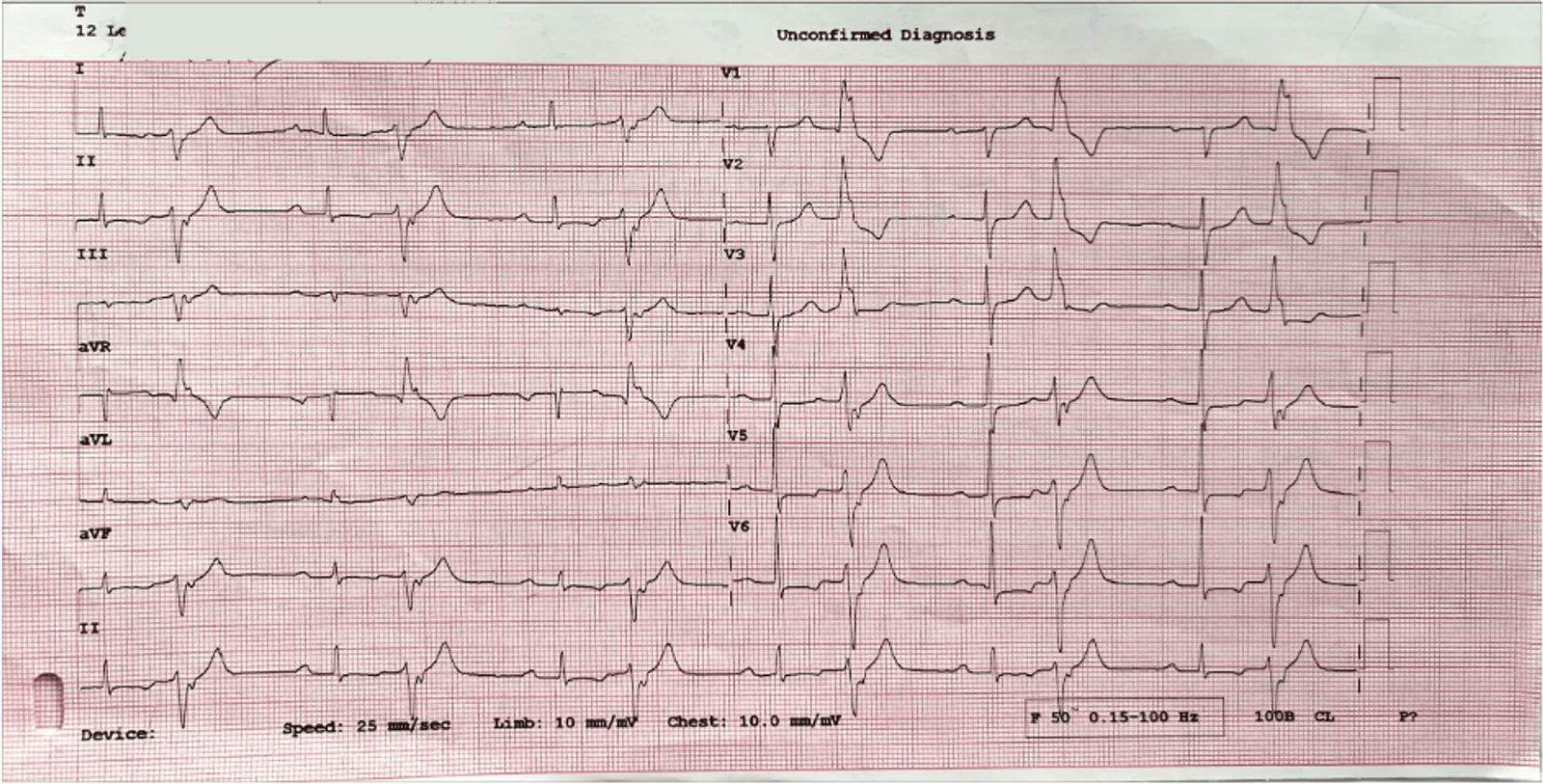
A 12-lead electrocardiography showing sinus rhythm with first degree AV block (PR 290 ms). ST-segment depression and biphasic T-waves in the inferior leads. Ventricular bigeminy with right bundle branch morphology is also present. The corrected QT interval is 462 ms.

**Figure 2:**
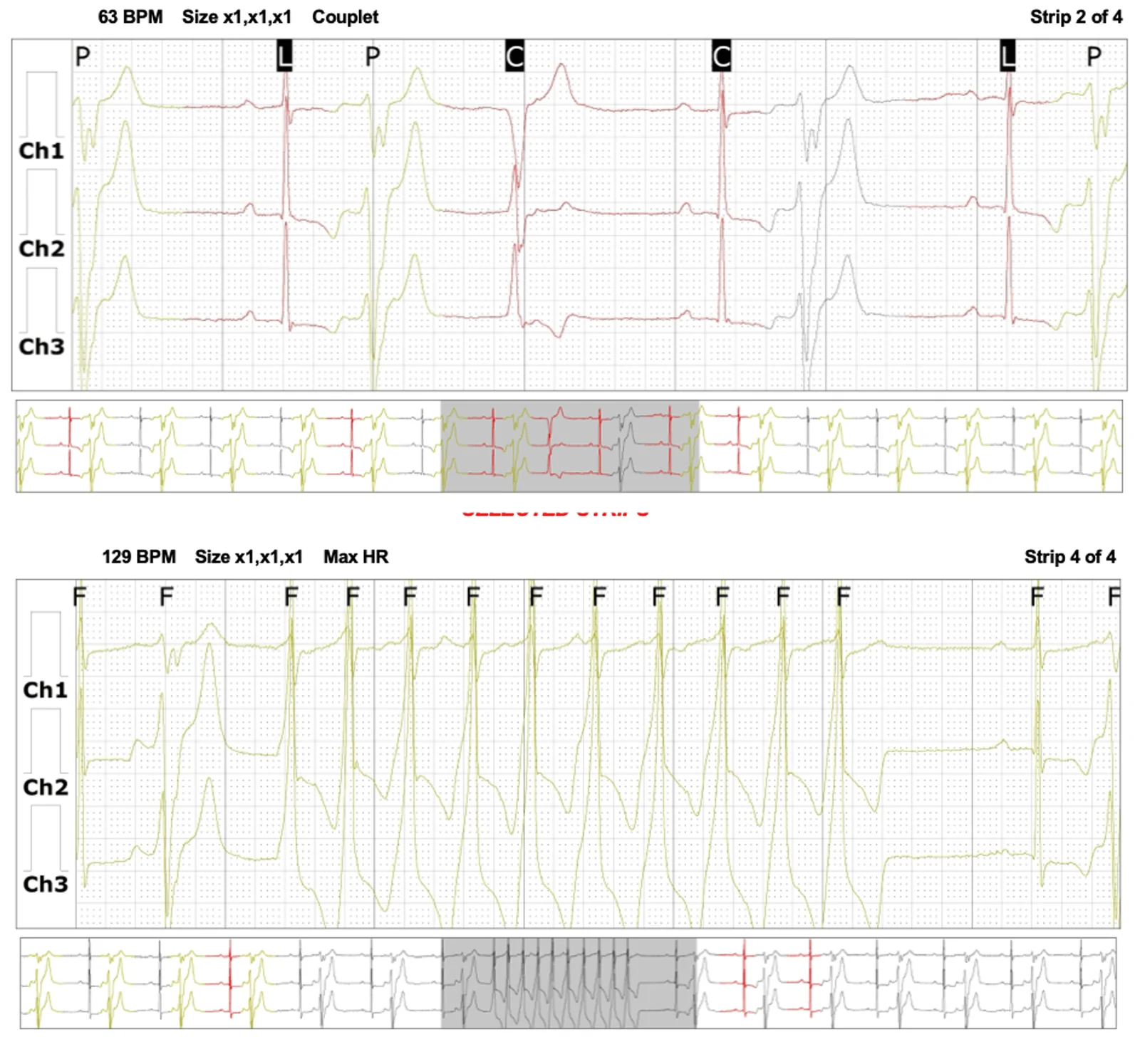
Representative Holter monitoring strips showing complex ventricular ectopy. The upper panel shows sinus rhythm with frequent premature ventricular complexes of two distinct morphologies, consistent with multifocal ventricular ectopy. The lower panel shows an episode of non-sustained monomorphic ventricular tachycardia consisting of 10 consecutive ventricular beats. The Holter recordings are displayed at a paper speed of 25 mm/s and a gain of 10 mm/mV. The three recorded channels are labeled and represent modified lead configurations approximately corresponding to standard leads I, II, and III.

Resting 12-lead electrocardiography demonstrated sinus rhythm with first-degree AV block (PR 290 ms), ST-segment depression, and biphasic T waves in the inferior leads, with ventricular bigeminy. The corrected QT interval was 462 ms (Bazett formula) (Figure 1). Twenty-four-hour Holter monitoring showed sinus rhythm with an average heart rate of 71 beats/min, a minimum heart rate of 38 beats/min, and a maximum heart rate of 129 beats/min. A high burden of ventricular ectopy was documented, with premature ventricular complexes accounting for 32% of all QRS complexes. The ectopy was polymorphic, and one episode of non-sustained monomorphic ventricular tachycardia lasting 10 beats was recorded (Figure 2).

**Figure 3:**
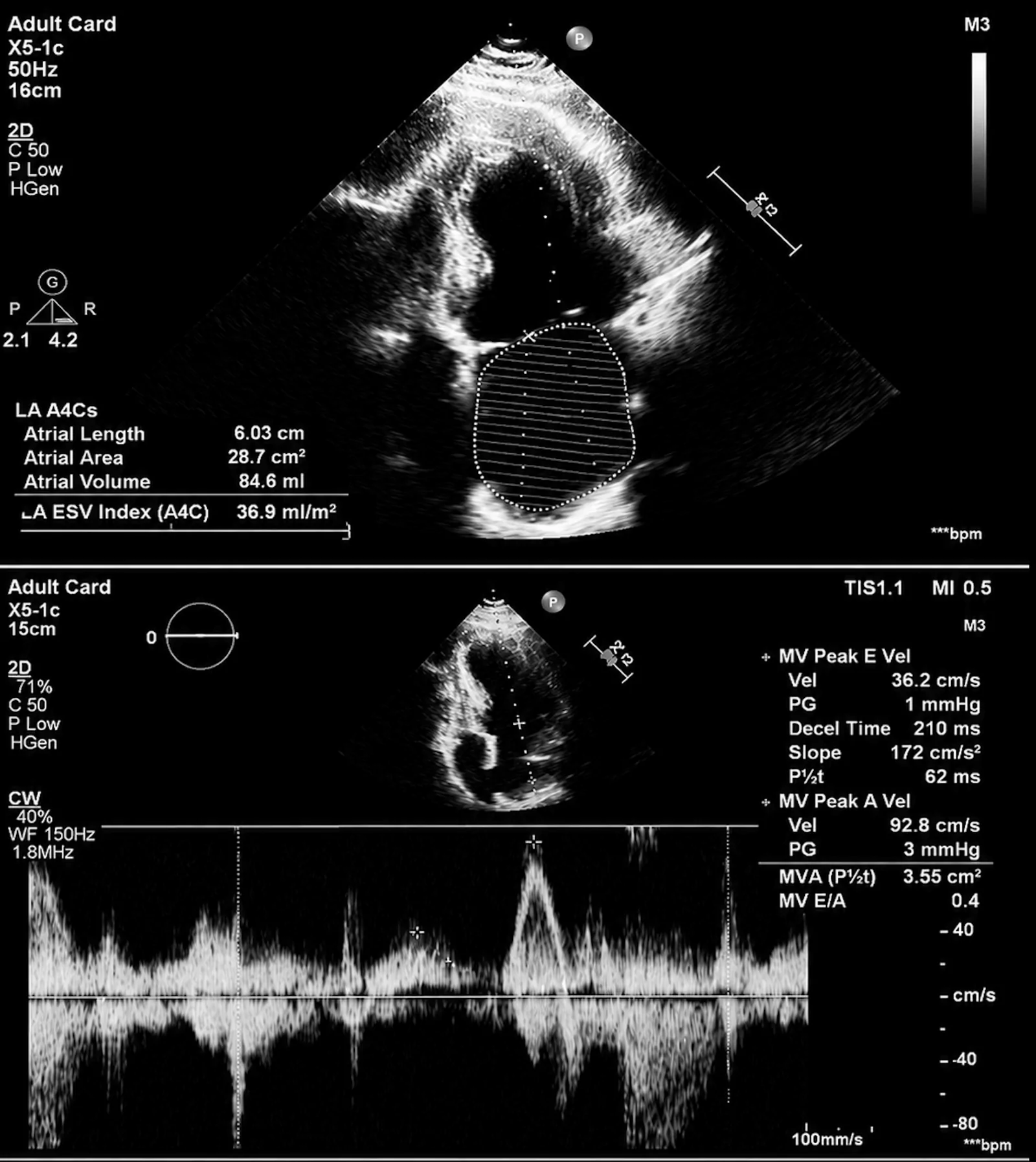
Transthoracic echocardiography showing left atrial volume index (LAVI) and mitral inflow velocities.

Transthoracic echocardiography demonstrated preserved left ventricular systolic function, with a left ventricular ejection fraction of 57%. The left ventricular end-diastolic diameter was 55 mm, and the end-diastolic and end-systolic volumes were 120 mL and 51 mL, respectively. Mild concentric left ventricular hypertrophy was present, with an interventricular septal thickness of 13 mm and posterior wall thickness of 12 mm. The left atrial volume index was 37 mL/m² (Figure 3). Right ventricular size and systolic function were preserved. The cardiac valves showed degenerative changes without hemodynamically significant stenosis or regurgitation. Estimated systolic pulmonary artery pressure was 35 mmHg.

Routine hematological and biochemical laboratory tests showed no clinically significant abnormalities relevant to the presenting cardiac condition (Table 1). Serological testing for *B. burgdorferi* was performed and demonstrated positive anti-*B. burgdorferi* IgG antibodies at 47 arb’U/mL (reference range: <10 negative, 10-15 borderline, >15 positive), while IgM antibodies were negative at 8.8 arb’U/mL (reference range: <18 negative, 18-22 borderline, >22 positive). The positive IgG result was interpreted as evidence of previous *Borrelia* exposure. A confirmatory immunoblot or second-tier enzyme immunoassay was not available.

**Table 1:** Laboratory findings. CRP: C-reactive protein; HDL-C: high-density lipoprotein cholesterol; LDL-C: low-density lipoprotein cholesterol; ALT: alanine aminotransferase; AST: aspartate aminotransferase; CK: creatine kinase; CK-MB: creatine kinase - MB isoenzyme; cTnI: cardiac troponin I; aPTT: activated partial thromboplastin time; INR: international normalized ratio; WBC: white blood cell count; RBC: red blood cell count; mg: milligram; g: gram; L: liter; mmol/L: millimoles per liter; µmol/L: micromoles per liter; U/L: units per liter; ng/mL: nanograms per milliliter; s: seconds

Laboratory parameter	Result	Reference range
C-reactive protein (CRP)	0.3 mg/L	0.0-5.0 mg/L
High-density lipoprotein cholesterol (HDL-C)	1.02 mmol/L	1.0-1.6 mmol/L
Low-density lipoprotein cholesterol (LDL-C)	2.53 mmol/L	0-2.6 mmol/L
Alanine aminotransferase (ALT)	31 U/L	10-49 U/L
Aspartate aminotransferase (AST)	21 U/L	0-34 U/L
Glucose	6.7 mmol/L	4.1-5.9 mmol/L
Creatine kinase (CK)	146 U/L	1-190 U/L
Creatine kinase-MB (CK-MB)	10 U/L	0-25 U/L
Serum creatinine	93 µmol/L	62-115 µmol/L
Triglycerides	1.64 mmol/L	0-1.7 mmol/L
Cardiac troponin I (cTnI)	<0.20 ng/mL	<0.20 ng/mL
Total cholesterol	4.3 mmol/L	2.70-5.20 mmol/L
Sodium	141 mmol/L	132-146 mmol/L
Potassium	4.8 mmol/L	3.50-5.50 mmol/L
Chloride	106 mmol/L	99-109 mmol/L
White blood cell count (WBC)	7.31 × 10⁹/L	3.60-10.50 × 10⁹/L
Hemoglobin	160 g/L	125-172 g/L
Red blood cell count (RBC)	5.33 × 10¹²/L	4.00-5.65 × 10¹²/L
Hematocrit	0.496 L/L	0.370-0.490 L/L
Platelet count	250 × 10⁹/L	140-440 × 10⁹/L

**Figure 4:**
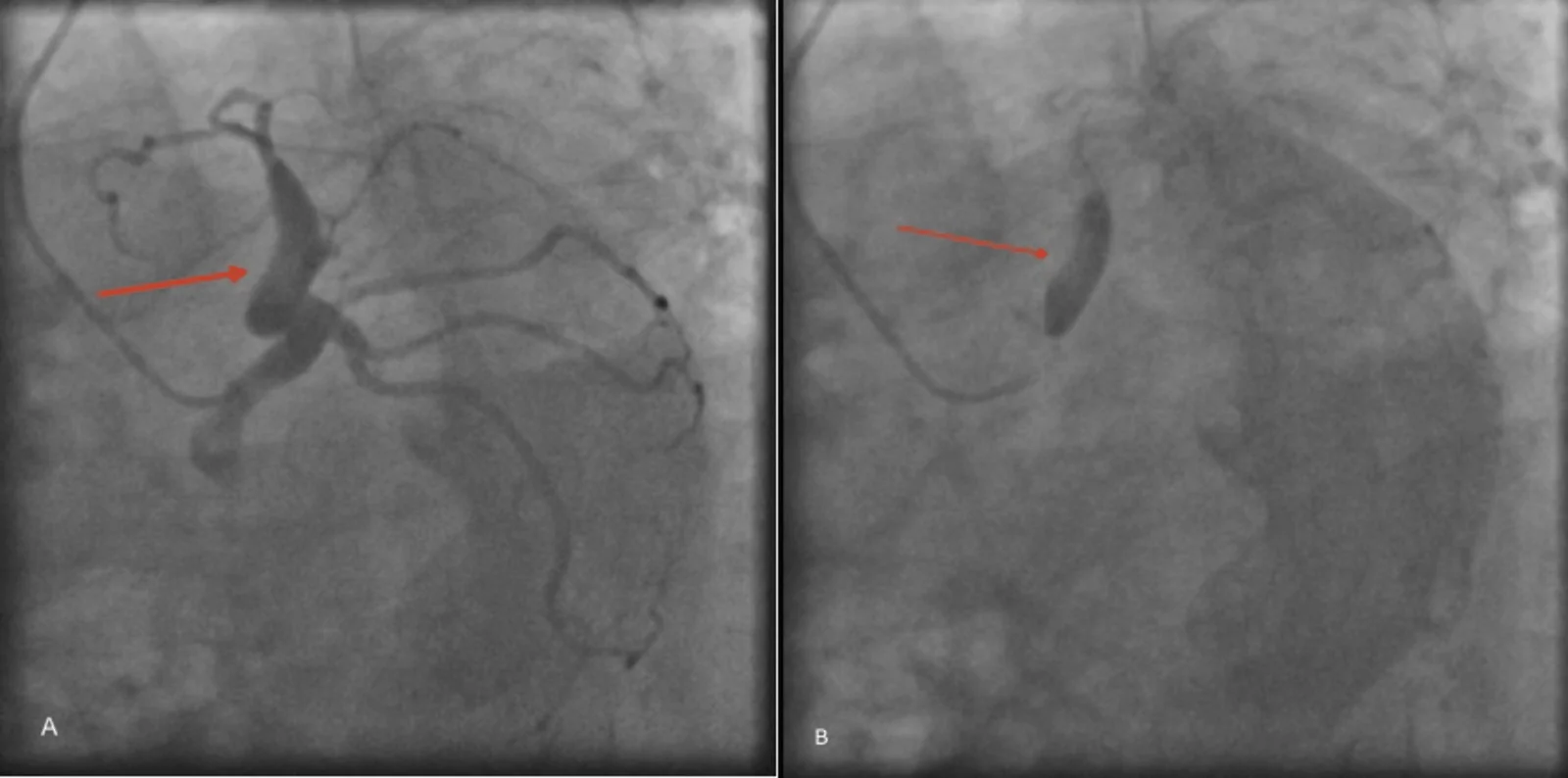
Left coronary angiography in LAO 40°/caudal 45° projection (Spider view) demonstrating ectasia of the left anterior descending artery (panel A) and persistent mural contrast retention 8 seconds after contrast injection (panel B). Red arrows indicate the ectatic segment. Contrast was administered using an ACIST CVi automated injection system, with a volume of 8 mL and a rise time of 3 seconds.

**Figure 5:**
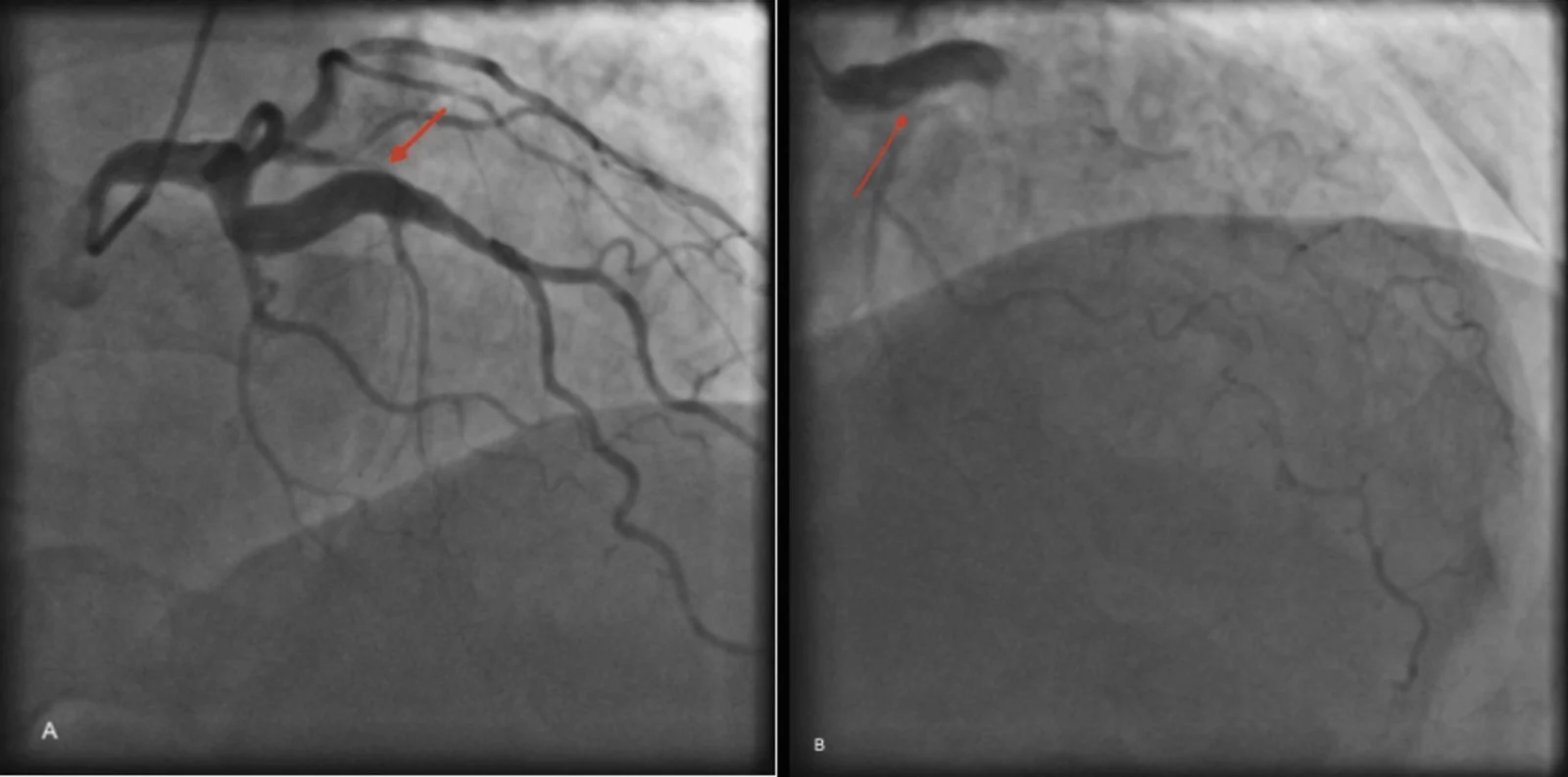
Left coronary angiography in RAO 20°/cranial 35° projection, showing ectasia of the left anterior descending artery (panel A) and persistent mural contrast retention 8 seconds after contrast injection (panel B). Contrast was administered using an ACIST CVi automated injection system with a volume of 8 mL and a rise time of 3 seconds. Red arrows indicate the ectatic segment.

**Figure 6:**
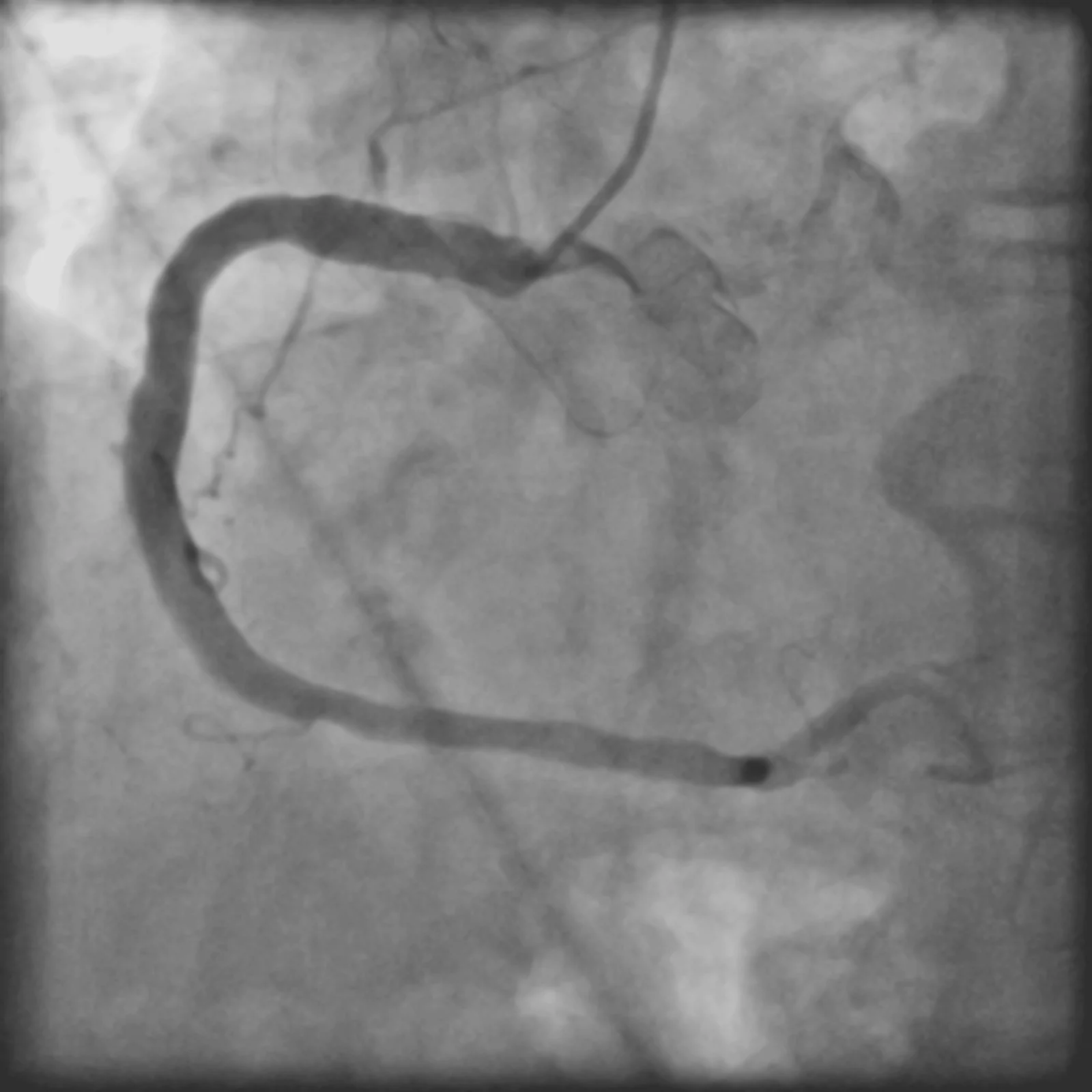
Right coronary angiography demonstrating coronary ectasia of the right coronary artery. Contrast was administered using an ACIST CVi automated injection system with a volume of 6 mL and a rise time of 2 seconds.

Given the exertional angina and the high burden of ventricular arrhythmia, invasive coronary angiography was performed. No significant obstructive epicardial coronary artery disease was identified. However, coronary artery ectasia was present, involving the left anterior descending and right coronary arteries. The maximal diameter of the ectatic segments was approximately 6.5 mm, exceeding 1.5 times the diameter of the corresponding reference segments. Coronary angiography was acquired at 10 frames per second. Contrast was administered using an ACIST CVi® automated injection system (ACIST Medical Systems, Inc., Eden Prairie, MN, USA), with 8 mL and a three-second rise time for the left coronary artery and 6 mL and a two-second rise time for the right coronary artery. Persistent mural contrast retention remained visible for 9.7 seconds after contrast injection, corresponding to approximately 97 frames (Figures 4-6).

Because of the coexistence of exertional anginal symptoms, persistent contrast retention within ectatic coronary segments, and frequent ventricular ectopy, ranolazine was started at a dose of 375 mg twice daily, with planned electrocardiographic and Holter monitoring. Antibiotic therapy with doxycycline 100 mg twice daily for 14 days was also initiated.

**Figure 7:**
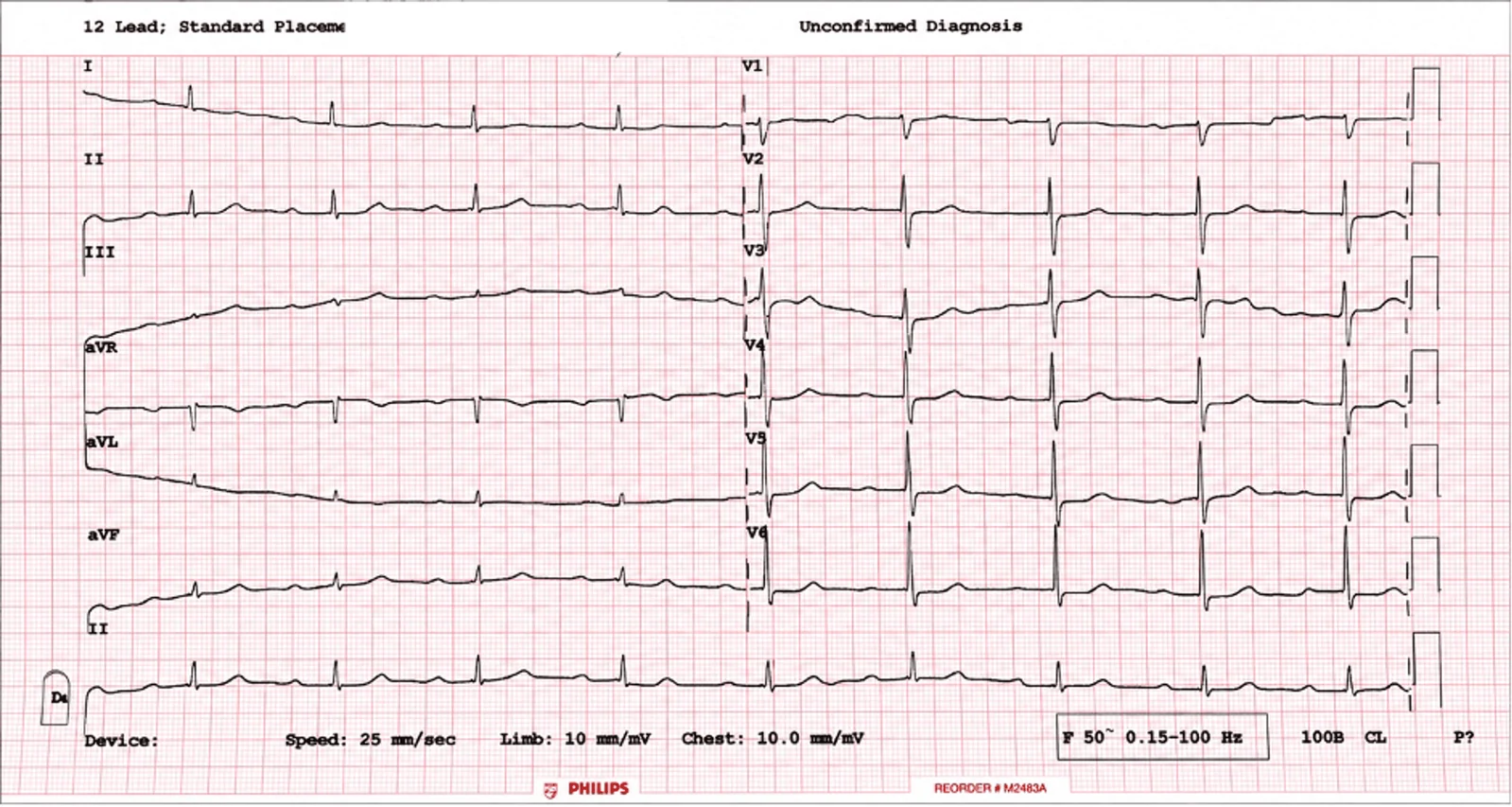
Electrocardiogram at one-month follow-up showing sinus rhythm without ischemic changes and without ventricular ectopy, with a QTc interval of 449 ms.

At one-month follow-up, the patient reported marked improvement in chest discomfort and palpitations, improved tolerance for routine physical activities, and expressed satisfaction with the clinical improvement following treatment. Repeat electrocardiography showed sinus rhythm and first-degree AV block (PR 290 ms), without ischemic changes, and the QTc interval was 449 ms (Figure 7). Control Holter monitoring demonstrated a substantial reduction in premature ventricular complexes, from 32% to less than 5% of all QRS complexes, with no further episodes of non-sustained ventricular tachycardia. At six-month follow-up, the patient remained clinically stable, with sustained improvement in symptoms and no reported recurrence of symptoms or other adverse clinical events. Ranolazine was continued throughout the six-month follow-up period and was well tolerated, with no reported treatment-related adverse effects. Follow-up echocardiography showed no clinically relevant change. A structured clinical timeline has been presented in Table 2.

**Table 2:** Structured clinical timeline. ECG: electrocardiography; PVC: premature ventricular complex; NSVT: non-sustained ventricular tachycardia, LVEF: left ventricular ejection fraction

Time	Clinical event
~8 months before admission	Tick bite followed by retrospectively reported erythematous lesion at the site of the bite; no antibiotic treatment
Following months	Progressive exertional chest discomfort, dyspnea, palpitations, reduced exercise tolerance
Admission	ECG: ventricular bigeminy and repolarization abnormalities; Holter: 32% PVC burden and 10-beat NSVT
During hospitalization	Positive *Borrelia burgdorferi* IgG, negative IgM; echocardiography with preserved LVEF
During hospitalization	Coronary angiography: multivessel coronary ectasia with marked contrast retention
After angiography	Ranolazine 375 mg twice daily and doxycycline 100 mg twice daily for 14 days initiated
1 month follow-up	Marked symptomatic improvement; PVC burden <5%; no further NSVT; QTc 449 ms
6 month follow-up	No recurrence of symptoms or adverse clinical events

## Discussion

The temporal relationship between the untreated tick bite and the subsequent cardiac presentation is clinically relevant in this case. Lyme carditis is reported to occur during the disseminated phase of Lyme disease, from several days to months after the initial infection, with most cases described between four days and seven months after disease onset [3,4]. Erythema migrans is the most common early manifestation, occurring in approximately 90% of cases. In our patient, a compatible local skin reaction was reported after the tick bite, although it was not medically documented at the time. Neurologic or cardiac manifestations are reported in approximately 10% of untreated cases [3,4].

Although atherosclerosis is the most common etiology of coronary artery ectasia in adults, non-atherosclerotic causes include congenital abnormalities, systemic inflammatory or connective tissue diseases, vasculitis, and infectious diseases [13]. Atherosclerotic remodeling remains an important competing explanation in this elderly patient with cardiovascular risk factors, and although no obstructive coronary artery disease was identified angiographically, non-obstructive atherosclerotic remodeling could not be excluded. In the context of the reported tick exposure, retrospectively described erythematous skin lesion, and positive *B. burgdorferi* IgG serology, a postinfectious coronary vascular sequela of Lyme borreliosis was considered. However, Lyme-associated coronary vascular injury remains an unproven hypothesis rather than an established cause of the observed coronary ectasia [13].

Coronary vascular involvement in association with Lyme borreliosis has been reported previously. Gasser et al. described coronary artery aneurysms in two patients with long-standing Lyme borreliosis, while Cuisset et al. subsequently reported a coronary aneurysm in a patient with Lyme disease treated with a covered stent [9,10]. More recently, Ahmed et al. described five patients with coronary artery ectasia and slow coronary flow whose anginal symptoms improved following ranolazine therapy [14]. Therefore, neither Lyme-associated coronary dilatation nor the use of ranolazine in coronary ectasia is unique to the present case. The potential incremental contribution of our case lies in the co-occurrence of prior tick exposure with *B. burgdorferi* IgG seroreactivity, multivessel coronary ectasia with marked contrast retention, and ventricular arrhythmia, followed by clinical and arrhythmic improvement after treatment.

Coronary ectasia with prolonged contrast retention provides a plausible link between the angiographic findings and the patient’s symptoms. Ectatic coronary segments with slow flow are associated with impaired distal perfusion, which may promote ischemia even in the absence of obstructive epicardial disease [15-17]. In this case, we hypothesize that these abnormal coronary flow dynamics may have been the reason for the patient’s anginal symptoms and ventricular ectopic activity; however, a causal relationship between myocardial ischemia and the ventricular arrhythmia cannot be established.

Therapeutic decision-making was influenced by several clinical factors. Beta-blockers were avoided because of the patient’s tendency toward bradycardia documented on Holter monitoring, while amiodarone and sotalol were considered unfavorable due to the prolonged QTc interval and the potential risk of further conduction disturbances in the setting of suspected Lyme-associated cardiac involvement. These considerations narrowed the available antiarrhythmic options and supported the use of an alternative therapeutic strategy.

Ranolazine was selected because of its antianginal effect and potential antiarrhythmic properties [18]. Clinical studies have demonstrated its efficacy in reducing angina, while additional data suggest a potential role in suppressing atrial and ventricular arrhythmias in selected patients [19,20]. In the present case, symptomatic improvement and a marked reduction in ventricular ectopy were observed during follow-up after completion of the doxycycline course and while the patient remained on ranolazine therapy, with no worsening of the QTc interval. The favorable clinical and Holter response supports the hypothesis that myocardial ischemia at the microvascular level contributed to the arrhythmic burden. However, causality cannot be definitively proven.

This case has several limitations. The proposed mechanism of Lyme-associated coronary vascular injury remains presumptive, as a prior inflammatory injury to the coronary vessel wall cannot be definitively demonstrated retrospectively once the acute process has resolved. Cardiac magnetic resonance imaging or inflammatory imaging could have provided additional information regarding myocardial inflammation or scar but would not have established that the observed coronary ectasia resulted from previous Lyme-associated vasculitis. Thus, the case supports a biologically plausible association rather than definitive proof of Lyme-induced coronary vasculitis. An additional limitation is that the available *B. burgdorferi* serology consisted of a positive quantitative IgG assay without confirmatory second-tier testing.

Despite these limitations, the case emphasizes several important clinical points. First, Lyme-associated cardiac involvement should be considered in patients with unexplained arrhythmias and a compatible exposure history, even in the absence of typical atrioventricular block. Second, persistent coronary contrast retention within ectatic coronary segments in patients with non-obstructive coronary arteries may represent a clinically meaningful substrate for both angina and ventricular arrhythmias. Third, ranolazine may be a useful therapeutic option in selected patients with angina and ventricular ectopy when beta-blockers or conventional antiarrhythmic drugs are limited by bradycardia, QT prolongation, or concern for conduction disease.

## Conclusions

In patients with coronary ectasia and prior tick exposure with *B. burgdorferi *IgG seroreactivity, Lyme-associated coronary vasculitis should be considered as a possible cause, although this mechanism remains rare and difficult to prove definitively. Coronary ectasia with contrast retention should be considered as a substrate for myocardial ischemia and ventricular arrhythmogenesis in patients with non-obstructive coronary disease. Ranolazine may represent a useful antianginal therapeutic option with potential antiarrhythmic effects, particularly in patients with limited tolerance for beta-blockers or conventional antiarrhythmic therapy.
